# Socioecological complexity in primate groups and its cognitive correlates

**DOI:** 10.1098/rstb.2021.0296

**Published:** 2022-09-26

**Authors:** Susanne Shultz, Robin I. M. Dunbar

**Affiliations:** ^1^ Department of Earth and Environmental Sciences, University of Manchester, Manchester, UK; ^2^ Department of Experimental Psychology, University of Oxford, Oxford, UK

**Keywords:** social complexity, social brain hypothesis, cultural evolution, brain size, encephalization, innovation

## Abstract

Characterizing non-human primate social complexity and its cognitive bases has proved challenging. Using principal component analyses, we show that primate social, ecological and reproductive behaviours condense into two components: *socioecological complexity* (including most social and ecological variables) and *reproductive cooperation* (comprising mainly a suite of behaviours associated with pairbonded monogamy). We contextualize these results using a meta-analysis of 44 published analyses of primate brain evolution. These studies yield two main consistent results: cognition, sociality and cooperative behaviours are associated with absolute brain volume, neocortex size and neocortex ratio, whereas diet composition and life history are consistently associated with relative brain size. We use a path analysis to evaluate the causal relationships among these variables, demonstrating that social group size is predicted by the neocortex, whereas ecological traits are predicted by the volume of brain structures other than the neocortex. That a range of social and technical behaviours covary, and are correlated with social group size and brain size, suggests that primate cognition has evolved along a continuum resulting in an increasingly flexible, domain-general capacity to solve a range of socioecological challenges culminating in a capacity for, and reliance on, innovation and social information use in the great apes and humans.

This article is part of the theme issue ‘Cognition, communication and social bonds in primates’.

## 1. Introduction

Primate evolution has been dominated by the emergence of intense sociality. A higher proportion of primate species live and forage in stable social groups than in any other mammalian order [1]. Although group-living primates reap clear benefits in terms of predation avoidance [2,3], they pay ecological and physiological costs associated with increased competition for access to resources [4,5]. Among the challenges faced by individuals within social groups are managing dominance relationships, coordinating activity schedules and making collective decisions about foraging routes, sleeping sites and patch residency times. It is important to appreciate that it is not group size *per se* that presents the challenge but rather that larger groups have more direct competition for access to resource patches, larger group spread, longer day ranges and larger home ranges [6–8]. In addition, relational complexity increases with group size as a result of individuals with differing energy budgets, resource holding potential, and reproductive and development states [9]. The result of this variance is that the more individuals there are in a group, and the more divergent their foraging strategies become, the more likely their time budgets will become desynchronized [10–14], and the more intense will be the stresses to which they are subjected if they stay together [4].

Individuals can, of course, mitigate high levels of resource competition with foraging adaptations, such as social information use, innovation, extractive foraging and tool use, that broaden their resource base, incorporate novel resources and allow them to exploit hard-to-access foods [15]. Managing social relationships can, however, be more challenging. Ensuring coordinated group travel, mitigating the negative impacts of dominance inequalities, reducing aggression, promoting tolerance, increasing social bonds and forming alliances to increase competitive ability or influence in group-level decisions are cognitively demanding [11,16–18]. Differences in activity scheduling preferences, for example, result in activity desynchrony, and is one of the principal mechanisms explaining sexual segregation in large herbivores [19–21]. In primates, a suite of behaviours that include grooming, policing, and coalition formation that emerge from, or facilitate, socially bonded groups may help manage conflicts of this kind [22]. As a result, anthropoid primate groups differ from most other mammals in the extent to which their social networks are highly structured [5,23]. In addition, a growing number of studies of monkeys, apes, humans and other mammals have demonstrated that the ability to negotiate relationships and be socially well embedded have direct health and fitness (i.e. fertility and longevity) benefits at the individual level, including such indices as adult [24–31] and young survival [25,32,33], immune function [34,35] and reproductive rates [36].

In addition to being intensely social, primates are also highly encephalized. The allometric slope between brain size and body size is steeper in primates than in all other mammals apart from cetaceans [37,38]. Why primates should invest so heavily in cognition, and why, within the primates, some species should do so much more than others, remains much debated [39]. Broadly speaking, five major adaptive arguments have been offered to explain brain evolution. These focus on the benefits of political strategizing (the Machiavellian [40] and Vygostskian [41] Intelligence hypotheses), the benefits of culturally transmitted learning [42–45], the demands of maintaining the social cohesion and stability of groups in order to solve ecological problems in a social context (the Social Brain hypothesis [4,46,47]), the ‘Expensive Brain’ hypothesis (brains are energetically extremely expensive, such that species that evolve large brains require energy-rich diets to afford the associated developmental and metabolic costs [48]) and the Dietary Challenge hypothesis (high quality, patchy food such as fruit and insects are cognitively more challenging to find and track in the landscape [49]). The Expensive Brain hypothesis points to consistent life-history shifts towards delayed maturity and expensive young associated with the evolution of large, metabolically costly brains [50]. A recent variation (the ‘Cognitive Buffering’ hypothesis) has suggested that seasonality and/or habitat quality can impose constraints on brain size, and enhanced cognitive abilities may help mitigate the resulting unpredictability of resources [51,52]. These five hypotheses divide naturally into three categories: one relates to the metabolic and energetic constraints associated with growing and supporting a large brain, a second reflects a capacity to cope with the spatial and temporal environmental variability and a third involves cognition that helps both mitigate the costs and exploit the advantages of living in groups (group augmentation).

Part of the reason for the continued debate over the drivers of primate brain evolution is that the cognitive challenges primates actually face remain poorly understood [53]. The battery of empirical cognition tests typically presented to primates (and other species) focuses on either ‘physical’ understanding of shape, quantity or causality, or the social understanding of social learning or theory of mind [54]. There are few causal links or evidence for how understanding of physical properties translates into foraging efficiency, foraging routes or diet choice. Although there are studies within a few species that demonstrate planning, anticipation and spatial memory [55–57], this approach needs to be more widely applied to determine how species differ in spatial and temporal cognition and how these relate to behavioural traits. Similarly, we have very limited evidence for embedded social cognition in primates apart from mentalizing (the capacity to understand others' intentions) [58] and the capacity to inhibit prepotent actions [18,59–62]. Both of these forms of explicitly social cognition correlate with brain size and with the size and bondedness of social groups [62]. Moreover, these data are currently available for only a relatively small number of unusually well-studied species. More importantly, the challenges associated with living in a social group are much broader than this narrow conception of ‘social intelligence’. In fact, rather than ‘social’ or ‘ecological’ intelligence most cognitive studies have focussed on executive function such as causal reasoning and oddity and displacement problem-solving [59,63,64] that are common to all domains of social and ecological life. Associative learning, as a cognitive process, is relevant to many of these tasks and is itself neither social nor physical [65]. In these cases, species differences in competence correlate with brain volume or the volume of core brain regions, such as the non-visual neocortex and the hippocampus [59]. A primary drawback with these cognitive indices is that less than two dozen of the 250 or so primate species have been tested on them.

A fundamental gap in our understanding of primate behavioural evolution is the link between cognition, ecology and social complexity. Relational versus organizational complexity, particularly among non-relatives, has been proposed as a unifying theme to characterize animal social complexity [9]. Cooperatively breeding species are characterized by highly defined and constrained roles (i.e. breeder and helper) with little opportunity for individualized strategies [9]. By contrast, species that exhibit relational complexity are characterized by differentiated social relationships that influence access to resources and mating. It is this second type of social complexity that probably holds the key to primate social cognition. In this paper, we begin with a suite of behaviours that are widely recognized as being central to primate socioecology and particularly relational complexity. These include social learning, diet, tool use, innovation, deception, coalition formation, collective action, policing, cooperative breeding, allocare, male care and indices of general intelligence. We build on previous studies which have compiled evidence that technical innovation and social learning are correlated with several brain size measures across taxa [42,66–69] by including a wider range of behaviours. The additional behaviours focus on cooperation and affiliation as fundamental aspects of primate sociality in addition to technological innovations [70–74]. Cooperative breeding has specifically been highlighted as a key evolutionary transition opening up human prosociality [75]. Collective action, such as range defence, that require coordination and synchrony are key to managing stable, bonded social groups (congregations) so as to bring group-level benefits in addition to individual benefits. While these behavioural competencies certainly mediate between cognition and sociality, and are sometimes treated as though they are proxies for underlying cognitive competencies, we actually have no idea what their cognitive bases are or whether they involve anything more than conventional executive function (planning, causal reasoning, learning).

We ask, first, whether these behaviours form natural clusters in which traits correlate closely across species and, if so, what characterizes these clusters. We hypothesize that if primate cognition is flexible and general, traits should covary and condense into aggregate indices, with socially complex species having more of these traits than less complex species. If these behaviours are associated with cognitive demands, we expect a correlation between socioecological complexity and brain size and architecture and performance on cognitive tasks. We then contextualize these results with previous studies of brain evolution in primates through meta-analysis of the seemingly conflicting results across previous studies. Finally, we use path analysis to examine the causal structure of these clusters in order to better understand how the variables relate to each other. Our aim is to differentiate relationships that reflect explicit selection relationships from those that involve evolutionary constraints (essentially the costs against which selection acts) or correlated consequences (windows of evolutionary opportunity).

## 2. Methods

### (a) Data

#### (i) Behavioural repertoire

We collated information on social, reproductive and foraging behaviours across species from the literature (electronic supplementary material, tables S1 and S3). These behaviours were social learning, extractive foraging, tool use, innovation (from [42]), deception [76], coalition formation, collective action (defined as joint range defence by more than one adult), policing, cooperative breeding, allocare and paternal care (see the electronic supplementary material). These behaviours were chosen because (i) they are frequently referred to as examples of cognitively complex behaviours in primates, (ii) they are well documented in the literature, and (iii) the first five behaviours have been used previously to evaluate an ecologically relevant cross-species index of primate general intelligence, *g_s_* [42]. Grooming rates and reconciliation were considered but rejected owing to issues with dichotomizing continuous variables and a lack of variance across species, respectively. In addition, grooming data are available only for a small subset of species, and this would radically reduce sample size for the analyses. The full dataset, complete definitions and list of references are provided in the electronic supplementary material, information. In contrast to *g_s_*, which uses the frequency of behavioural reporting, we assigned a dichotomous score for observed presence or absence for each behaviour. We chose to use dichotomized variables because (i) rates or frequencies are not appropriate for many of the behaviours, (ii) many of these behaviours are rare making rate or relative frequency estimates challenging, and (iii) detailed quantitative information on specific behaviours are only available from a small number of studies. By contrast, a presence/absence of dichotomy is easier to establish and should be less sensitive to research effort. Because rare social behaviours are likely to be under-represented in poorly studied taxa, we included only species with more than 25 published papers (the median value in a highly skewed distribution) that focus on behaviour and/or ecology. We excluded all species with missing data, resulting in a final sample of 129 species. As some data are reported at the genus level, we then reduced all data to 68 genera for the behavioural analyses by taking mean values across genus members.

#### (ii) Brain size

We used three sets of brain data: (i) total brain volume, neocortex volume, executive brain and neocortex ratio were taken from a compilation of histological and imaging datasets [68,77], and (ii) a larger but lower resolution endocranial volume dataset [78]. The gross measures were log_10_-transformed for all analyses. We report multiple measures of brain size including: relative brain size (brain volume controlling for body size), absolute brain volume, neocortex volume and neocortex ratio. The complement of species varied between brain size measures such that the degrees of freedom vary in the final analyses.

#### (iii) Group size and diet breadth

Because group size is invariably Poisson-distributed within species, we used the geometric mean from the reported range of social or community group sizes documented for each species, taken from [79,80]. These means were log_10_-transformed for all analyses. Estimates for primate diet breadth were taken from [42]. We note that a key aspect of primate social structure is the number of adult females in a group [81]. However, as the number of females in primate groups is highly correlated with group size across the full range of primate species [82], we use total group size in all analyses.

#### (iv) Cognition measures

We collated estimates of primate cognitive performance from two published sources. Deaner *et al*.'s [64] general empirical intelligence measure (which we here term *g_D_*, based on an aggregate of task performance in the laboratory) and Reader *et al*.'s [42] ecological ‘*g_s_*’ (derived from ecologically relevant behaviours). We reduced the latter to the genus level by taking the average score across genus members.

#### (v) Phylogeny

We downloaded a consensus tree from the 10kTrees site (v. 3, [83]).

### (b) Statistical analyses

We undertake four separate analyses: a principal components analysis (PCA) of social complexity, a multiple regression to evaluate the relationships between the factors identified by this analysis and cognitive ability, a quantitative synthesis of brain size evolution studies and a path analysis to unpack causal relationships. We detail the methods for each separately.

#### (i) Principal components analysis

To determine whether the presence of different behaviours covaried, we first evaluated the explanatory power for multiple factors, and the optimal number of factors to retain, using eigenvalues in a scree plot and a parallel analysis (electronic supplementary material, figure S1*a*,*b*). We then used a *polychoric* PCA to identify the components because this infers a latent Pearson correlation structure and thus is appropriate for dichotomous variables [84]. This was executed in the R *psych* package [85], using the *polychoric* function to identify the correlation matrix; we then identified principal components using the *principal* function, with a ‘varimax’ rotation; and finally we used the *factor.scores* function to extract species component scores. Because the resulting scores were correlated with the number of publications for each species, we corrected these scores by taking the residuals from a phylogenetic regression of the behavioural score against log_10_ publications. Although the use of residuals from OLS regression is potentially problematic [86], it is the most common way of controlling for research effort. However, to assess the robustness of this measure, we also (i) calculated a ratio of score to publications (we standardized the scores to be positive and divided by the log_10_ number of publications derived from searching Google Scholar), (ii) controlled for research effort in the subsequent analyses by including research effort as a covariate and (iii) analysed the behavioural scores without correcting for research effort. Results for these other measures are reported in electronic supplementary material, table S3.

#### (ii) Phylogenetic generalized least squares regression

To evaluate the relationships between the behavioural scores, brain and group size, we used phylogenetic generalized least squares (PGLS) with optimized lambda in the R package *ape* [87]. We used genera means for all cognitive analyses because *g_D_* is reported at the genus level. Finally, to evaluate how our measures of behavioural repertoires are associated with *g_D_*, *g_S_* and the ecological measures we also used PGLS.

#### (iii) Synthesis of comparative brain studies

To evaluate how consistent the relationships are between brain size measures, ecology, sociality and life-history measures, we compiled a comprehensive database of primate brain size studies (see the electronic supplementary material for search terms). We collated information for each brain dataset: species number, analytical approach (i.e. whether phylogenetically controlled, univariate versus multivariate model, brain size measure used), and *p*-values for relationships. Where multiple analyses were presented in a single paper, we chose the most conservative result. For example, where univariate and multivariate models were presented, we used the *p*-value associated with brain size from the latter. Similarly, where models with and without phylogenetic control were both presented, we use the *p*-value from the former. Where papers presented multiple measures for a single category (e.g. ecological variables), we used all reported measures. The variables we use and their sources are defined in the electronic supplementary material. These results are then subjected to meta-analysis.

Meta-analysis combines data from a number of studies that test the same underlying hypothesis, weighting the statistical parameter by the sample size. The statistical approach and the information provided vary widely across the studies in our sample. As not all reported variance measures, it was not possible to calculate effect sizes across all papers. However, most papers reported *p*-values associated with their analyses, although in some cases *p*-values for non-significant findings were not reported. We combined study *p*-values using the *Z*-transformation (Stouffer's method [88]), weighted by the sample size of the study as follows:

$$\frac{\sum_{i=1}^{k}\omega_{i}Z_{i}}{\sqrt{\sum_{i=1}^{k}\omega_{i}^{2}}}\;,$$

where *ω_i_* is the weight associated with each of the *i* = *k* studies. Here, we use the square root of the number of taxa as the weight. *Z_i_* is the *Z*-score for the *i*th study, calculated from the reported *p*-value. Since the combined test statistic can be inflated owing to reporting bias, and we cannot control this, we also report the median *p*-value reported for each study in order to evaluate the consistency of the results [89].

#### (iv) Path analysis

To show how path analysis can disentangle alternative interpretations of the evolution of primate brain size, we reanalysed endocranial [90] and neocortex data [91] using a modified path analysis approach. We use Powell *et al*.'s ecological data [90] rather than the dataset published by DeCasiens *et al*. [92], as the diet data from the latter is problematic both in terms of agreement with other published data and consistency of categorical definitions (see the electronic supplementary material). We also analysed a subset of these data for which actual neocortex data are available [91] so as to compare the resulting models.

For each variable, we used multi-model dredging using the *dredge* function in package MuMIn [93] to select the best subset of candidate models based on Akaike information criterion corrected for small sample sizes (AICc) and model weight. The *dredge* function permutes all possible PGLS models. As identifying dependent and independent factors in morphology, ecology and behaviour are difficult to disentangle, we ran models where each factor was predicted by all other factors. We present the associations identified by the best fit model as well as cumulative model weight for each variable.

## 3. Results

### (a) Identifying functional subsystems

**Figure 1.**
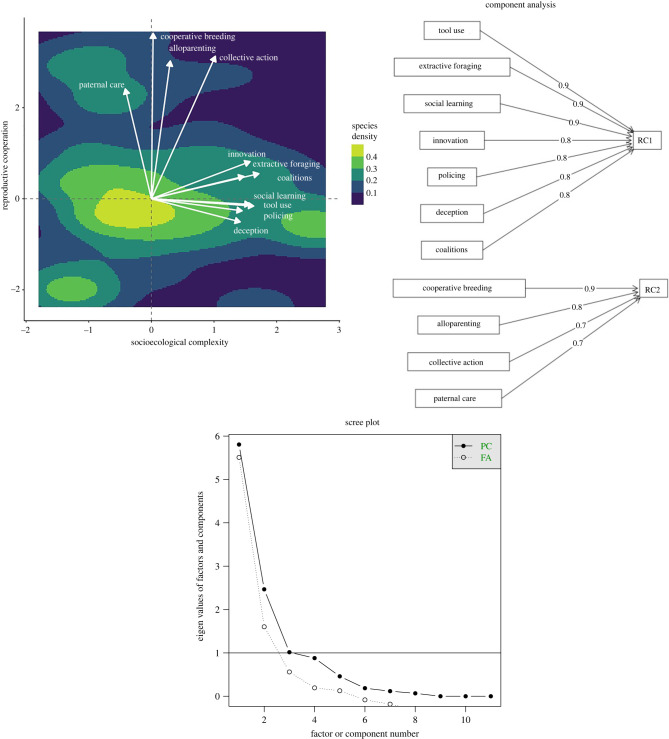
Polychoric PCA results for behaviours (main image), which is supported by the factor plot (upper right) and the scree plot of both factors and principal components (lower right). The first component (socioecological complexity) explains 52% of the variation and is associated with innovation, deception, social learning, coalitions, policing and extractive foraging. The second component (reproductive cooperation) explains a further 24% of the variance and is associated with cooperative breeding, paternal care, alloparenting and collective action (joint range defence). The PCA plot is coloured by the density of species scores on the two axes, with lighter colours representing higher species density. The loading of each factor is shown by white arrows, with their length proportional to the weight.

The polychoric PCA identifies two components: the first includes innovation, social learning, extractive foraging, tool use, deception, coalitions and policing (explaining 52% of the variance) and the second includes the reproductive behaviours: allomothering, cooperative breeding and paternal care, and collective action (which explains 24% of the variance) (figure 1; electronic supplementary material, table S1). These two components together explain 76% of the overall variance. We label the first factor *socioecological complexity*, as it incorporates ecologically and socially relevant behaviours, and the second *reproductive cooperation*, which identifies pairbonded, and particularly cooperative breeding, species.

To explore the interrelations between these behavioural indices, the measures of intelligence and brain size we first ran bivariate correlations, and then used these to build multivariate models.

At the genus level, the *socioecological complexity* index was significantly correlated with the two indices of general intelligence (*g_D_*–*g_s_*: *λ* = 0.0, *t*_16_ = 3.55, *p* = 0.003, $r_{\mathrm{adj}}^{2}=0.41$; *g_D_*–*socioecological complexity*: *λ* = 0.17, *t*_23_ = 2.13, *p* = 0.04, $r_{\mathrm{adj}}^{2}=0.13$; *g_s_*–*socioecological complexity*: *λ* = 0.67, *t*_27_ = 2.32, *p* = 0.03, $r_{\mathrm{adj}}^{2}=0.14$). Although *g_s_* and *socioecological complexity* have several measures in common, the fact that *r*^2^ is similar for both intelligence indices suggests that the correlation with *g_s_* cannot be entirely explained by this. *Reproductive cooperation* was not correlated with *g_D_* (*λ* = 0.09, *t*_23_ = 2.10, *p* = 0.05, $r_{\mathrm{adj}}^{2}=0.12$) or *g_s_* (*λ* = 0.71, *t*_23_ = −0.84, *p* = 0.41, $r_{\mathrm{adj}}^{2}=-0.01$), suggesting that maintaining pairbonded relationships is less cognitively demanding, at least in primates.

Both *g_s_* (*λ* = 0.97, beta 1.67 ± 0.47, *t*_25_ = 3.54, *p* = 0.002, $r_{\mathrm{adj}}^{2}=0.31$) and *socioecological complexity* (*λ* = 0.0, beta 0.76 ± 0.24, *t*_48_ = 3.13, *p* = 0.003, $r_{\mathrm{adj}}^{2}=0.15$) were positively correlated with mean reported group size for each genus. *Socioecological complexity* was not associated with diet breadth (*λ* = 0.09, beta 0.16 ± 0.10, *t*_53_ = 1.53, *p* = 0.13, $r_{\mathrm{adj}}^{2}=0.02$). *Reproductive cooperation* was not significantly associated with group size (*λ* = 0.99, beta 0.03 ± 0.38, *t*_48_ = 0.07, *p* = 0.95, $r_{\mathrm{adj}}^{2}=-0.02$) or with diet breadth (*λ* = 0.93, *β* − 0.09 ± 0.11, *t*_53_ = −0.86, *p* = 0.39, $r_{\mathrm{adj}}^{2}=0.004$).

**Figure 2.**
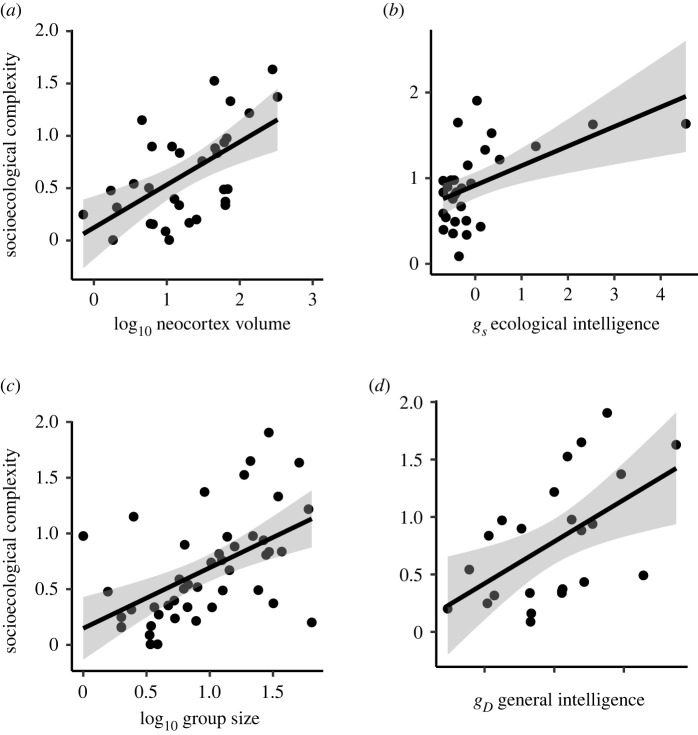
Socioecological complexity is positively associated with (*a*) neocortex ratio, (*b*) *g_s_*, (*c*) group size, and (*d*) the composite general intelligence *g_D_* score based on laboratory cognitive tests from Deaner *et al*. [64]. Only genus means are presented since Deaner *et al*. conducted analyses at the genus level.

All the examined brain size measures, apart from relative brain size (i.e. controlling for body size), were positively associated with *socioecological complexity*, *g_s_* and *g_D_* (table 1 and figure 2). By contrast, *reproductive cooperation* was negatively associated with endocranial volume, and neocortex volume but not correlated with neocortex ratio.

**Table 1.** Phylogenetic generalized least square models of brain size measures and different cognitive/behavioural indices. (ECV stands for endocranial volume. Ecological intelligence and general empirical intelligence derived from previous studies [42,64,66,67]. Note we use the inverse of *g* so that all metrics scale in the same direction. Socioecological complexity and reproductive cooperation are derived here.) *p ≤ 0.05, **p ≤ 0.01, ***p ≤ 0.001.

	model	brain measure (d.f.)	estimate	*t*	Pr(>|*t*|)
ecological intelligence *g_s_*	1	log ECV	1.23 ± 1.48	0.83	0.413
		log body size (2,27)	0.04 ± 1.12	0.03	0.975
	2	log ECV (1,28)	1.28 ± 0.33	3.89	0.001***
	3	log neocortex (1,20)	1.13 ± 0.37	3.02	0.007**
	4	neocortex ratio (1,20)	1.59 ± 0.46	3.43	0.003**
general empirical intelligence *g_D_*	1	log ECV	0.01 ± 0.87	−0.01	0.990
		log body size (2,23)	0.77 ± 0.678	1.14	0.268
	2	log ECV (1,24)	0.98 ± 0.15	6.40	<0.001***
	3	log neocortex (1,17)	0.77 ± 0.17	4.53	<0.001***
	4	neocortex ratio (1,17)	−0.64 ± 0.22	2.94	0.009**
socioecological complexity	1	log ECV	0.45 ± 0.56	0.80	0.426
		log body size (2,47)	−0.01 ± 0.44	−0.03	0.979
	2	log ECV (1,48)	0.44 ± 0.11	4.01	<0.001***
	3	log neocortex (1,30)	0.41 ± 0.10	3.97	<0.001***
	4	neocortex ratio (1,30)	0.42 ± 0.09	4.50	<0.001***
reproductive cooperation	1	log ECV	−1.59 ± 0.85	−1.88	0.067
		log body size (2,47)	0.77 ± 0.61	1.25	0.216
	2	log ECV (1,48)	−0.57 ± 0.22	−2.57	0.013*
	3	log neocortex (1,30)	−0.50 ± 0.19	−2.65	0.013*
	4	neocortex ratio (1,30)	−0.41 ± 0.24	−1.72	0.095

### (b) Synthesis of comparative brain studies

**Figure 3.**
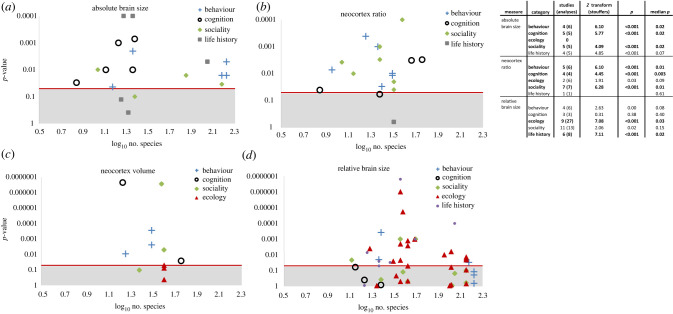
The relationship between log_10_-transformed number of species sampled and the significance (*p*-value) of the correlation between each brain index and the five behavioural variables (behaviour, cognition, social organization, life history and ecology) for each of the indices of brain size. (*a*) Absolute brain volume (cc), (*b*) neocortex ratio, (*c*) absolute neocortex volume (cc), and (*d*) relative brain size (residual of brain volume from the brain/body mass regression). Significant relationships fall above the horizontal line at *p* = 0.05. The studies that fall into the shaded areas found non-significant relationships between that brain measure and their trait of interest. Symbols correspond to the brain measures used. The statistics from a meta-analysis of *p*-values across studies for the three main brain indices are shown in the table. (Online version in colour.)

In this section, we summarize the results obtained by all the studies that have evaluated the relationships between brain size, socio-demographic variables, cognitive variables and a range of ecological traits (including diet, range size, strata use and activity patterns) (figure 3; electronic supplementary material, table S5). We located 44 such published studies. These studies have used a variety of behavioural, ecological and cognitive measures with different samples of species. The most common brain size measures used were either relative brain size (i.e. the analyses controlled for body size) or neocortex ratio (the ratio of neocortex volume to the rest of the brain). Some studies also use absolute brain or neocortex volume, but these measures were used more often in comparative cognition rather than ecological studies. We grouped these analyses into four categories ‘behaviour and cognition’, ‘social organization’, ‘ecology’ and ‘life history’. The details for the specific measures are in the electronic supplementary material. Briefly, ‘behaviour and cognition’ included studies of empirical cognition, specific social behaviours such as deception, agonism and coalitions as well as ‘field derived’ cognition such as innovations and social learning. Social organization studies focus on group size, social network structure and categorical descriptions of social organization (e.g. multi-male/harem/pair living). Life-history studies focus on age at maturity, longevity, basal metabolic rate, sexual dimorphism, etc. Finally, ecological studies focus on diet and home range characteristics.

In terms of ecology, small relative brain size (either as a residual or in models controlling for body size) is associated with a folivorous diet (6 out of 7 studies that included this variable), but not, it seems, with per cent fruit in the diet (1 out of 2), home range size (1 out of 5), strata use (1 out of 3) or activity pattern (0 out of 3) (figure 3). Although the sample sizes are small in the latter case, the pattern is generally consistent across the four ecological indices. Relative brain size is a rather better predictor of life-history measures such as basal metabolic rate (2 out of 3) and longevity (2 out of 3), but sample sizes are again small. By contrast, ecological measures are not associated with absolute brain size (0 out of 2) or neocortex ratio (1 out of 5). There are no evaluations of how ecology relates to either absolute or relative neocortex size.

By contrast, species' performances on cognitive tasks, cultural behaviours and innovations are consistently related to overall brain size (8 out of 9), with the best predictor of behavioural and cognitive competence being neocortex size (8 out of 8 studies; figure 3; electronic supplementary material, table S4). Notably, there are a number of studies that report significant correlations between cognition and neocortex ratio (6 out of 6), but few do so with relative brain size (3 out of 9) (although none directly compare these two measures). The one case where absolute brain size is not associated with cognitive behaviour is a study of non-technical innovations [66] that are not socially transmitted. Similarly, social measures are generally associated with absolute brain size (3 out of 4), the size of the neocortex or its subregions (4 out of 6) and neocortex ratio (6 out of 6). Unfortunately, very few studies have evaluated the relationship between non-visual cortex (i.e. neocortex minus the visual system in the occipital lobe, a brain region not specifically involved in social or ecological information processing) and social or cognitive behaviours, but those few that have done so have found a decidedly strong relationship (2 out of 2). Sociality is, however, less consistently associated with relative brain size (7 out of 15).

A meta-analysis of the data highlights the consistency in these results (figure 3). It is, of course, possible that the resulting statistic may be inflated by the lack of reporting of non-significant findings. In addition, the non-independence in brain datasets across some (but not all) studies means that the meta-analysis should be interpreted with caution. Nonetheless, the broad pattern shown in the electronic supplementary material, table S2 is remarkably consistent and supports the findings from the path analyses (see below): absolute brain size and neocortex ratio correlate consistently and strongly with social and cognitive variables, whereas relative brain size correlates consistently, though less strongly, with ecology and life-history variables.

### (c) Path analysis

**Figure 4.**
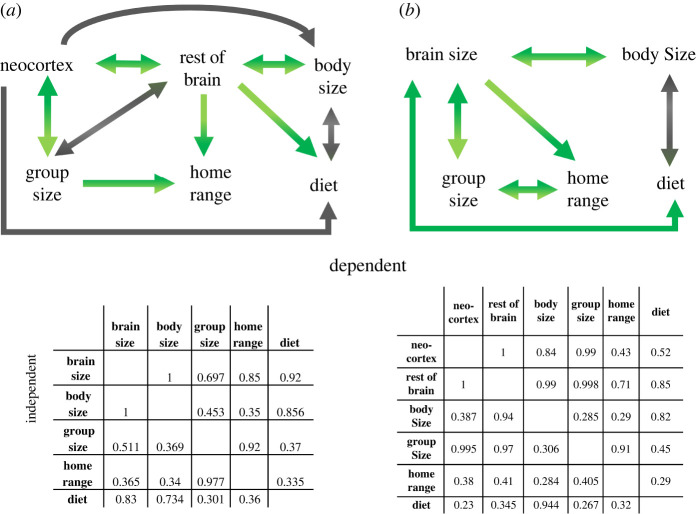
Arrows highlight associations identified with ‘best fit’ models from dredging a PGLS model with each term sequentially modelled as the dependent factor for (*a*) endocranial volume and (*b*) neocortex and rest of brain. Green arrows show a significant positive association and grey arrows show a significant negative association. No arrow (or no arrow tip) indicates no relationship identified in the best fit model. The matrix shows cumulative model weight for each independent variable (left-hand row) predicting each dependent (top row). Model average results with confidence intervals are given in the electronic supplementary material. (Online version in colour.)

To disentangle the inter-relationships between the various factors, we ran a path analysis on two sets of data: the first uses endocranial volume as the brain size measure. The second uses a smaller dataset with neocortex and rest of brain volume analysed separately. The models that best explained whole brain measures suggest that brain size is best predicted by increasing body size, group size and frugivory, whereas neocortex size is best predicted by group size and rest of brain (figure 4). Frugivory is positively predicted by the rest of brain volume but negatively with neocortex volume. Group size is predicted by a positive association with brain size and neocortex volume but is negatively associated with the rest of brain volume. Home range for both models is positively associated with group size and brain or the rest of brain volume.

## 4. Discussion

Our analyses highlight three explicit points. First, the aspects of primate social behaviour that we evaluate here can be condensed to two separate clusters that are underpinned by different cognitive and neural mechanisms, both of which have direct implications for fitness. One focusses on traits associated with reproductive cooperation; the other on traits associated with socioecological complexity and relate to the challenges of living in social groups (some of which relate to social coordination and others to foraging decisions). One important correlate is that socioecological complexity increases with group size. Second, socioecological complexity, empirically measured general intelligence [63] and ecological intelligence [66] are all strongly correlated with each other and with absolute brain measures, but not with relative brain size. By contrast, our aggregate measure of reproductive cooperation was not associated with group size or any brain size measures. Third, the results explain the apparently contradictory findings across primate comparative brain studies. Studies evaluating the relationship between cognitively relevant behaviours such as performance on empirical tasks, prosocial behaviours, tool use and social learning typically find consistent relationships with absolute brain measures or neocortex ratio than with relative brain size [22,42,59,60,66,94]. By contrast, studies evaluating the relationship between energetic constraints (i.e. diet, life history, home range size) find more consistent relationships with relative brain size than with absolute measures [92,94,95]. This is supported by the path analyses, which highlight similar relationships between neocortex and sociality and the rest of brain with diet and home range size. Thus, overall brain size, relative forebrain size and number of neurons are most strongly associated with cognition and with social traits, whereas the relative size of the brain is most strongly associated with diet quality, life history and development.

Together, this separation between the ‘cognitive’ and the ‘energetic’ (i) explains why previous analyses have found evidence to support seemingly contradictory positions, and (ii) underscores the fact that primate absolute brain size and architecture consistently predict social and cognitive traits. This is a timely reminder that stable, bonded groups of the kind characteristic of primates do not come for free: they are socially and cognitively expensive to maintain because the pressures promoting fragmentation in mammal groups are extremely high [4,62]. In addition, brains are nutritionally expensive and require dietary strategies that enable them to be both evolved and maintained.

The contrast between the two behavioural complexes is, at root, a contrast between species that have evolved pairbonded monogamy and those that have evolved stable, bonded social groups [62]. The demands of these two systems are very different for two complementary reasons. One is that monogamy can only evolve in habitats of low predation risk, where animals can afford to live in very small social groups. In the absence of factors influencing male mating strategies, females would do best to live and forage alone (in the company of their offspring) [4]. The other is that living in large stable congregations (as opposed to aggregations or fission-fusion structures) is demanding both in terms of building consensus, making collective decisions, and mitigating the effects of resource competition across individuals with differing energy budgets and resource holding potential [9]. These decisions are made in the context of the nutrient demands of growing and maintaining a large brain. Managing social relationships requires highly specialized cognitive skills such as mentalizing and the capacity to inhibit prepotent actions [18,62] that are neurophysiologically extremely costly [62,96], together with a range of generic cognitive skills (causal reasoning, analogical reasoning, one-trial inferential learning) that depend on brain regions (notably Brodman Area BA10) that are unique to the anthropoid primates [61].

Our results, which show that capacity for socioecologically complex behaviours covary, together with previous evidence for a primate ‘*g*’ [42,64], suggest that much of primate cognition is better described as domain general. Large-brained species perform well on associative learning as well as other cognitive tasks, are behaviourally, and hence culturally, complex, technologically sophisticated, have broad social repertoires and live in larger groups. Unlike perceptual capacities (which are usually highly specialized), cognitive capacities are about information processing and decision-making, and are rarely so specialized—and especially so in species that are behaviourally flexible. This makes it difficult to undertake comparative analyses that isolate out functional questions about selection. However, the fact that many of the core primate cognitive specialities (including causal reasoning, analogical reasoning and the capacity to inhibit behaviour) play a crucial role in both social and foraging tasks does not mean that both of these domains played an equal role in selecting for these abilities. When multiple causes are involved, it is statistically unlikely that they will have exerted identical selection pressures on the dependent variable. A more plausible explanation is that one is the original selection pressure for a cognitive upgrade that was subsequently exploited in other domains.

The question is whether the additional cognitive processing capacity represented by large brains evolved to allow animals to solve social problems (so as to live in large groups), with these cognitive skills later exapted to facilitate smarter foraging (e.g. tool use and extractive foraging), or evolved to allow animals to forage more effectively in challenging environments, which then allowed them to live in large groups. In primates, stable foraging groups emerged concurrently with the shift from nocturnal to diurnal activity patterns [71]. Tool use and extractive foraging, by contrast, is seen in only a small number of lineages (cercopithecines, great apes), most of whom have relatively recent evolutionary origins. Thus, there is clear evidence that social foraging is an earlier evolutionary response than sophisticated foraging. This suggests that the first option would thus seem to provide a more coherent sequence: living in large groups is a solution to the problem of occupying predator-risky habitats, with large brains the solution for the cognitive skills needed to maintain the cohesion and coherence of large groups, while enhanced foraging skills are necessary to maintain these calorie-hungry brains.

The consistent message from primate cognition studies is that the best predictors of cognitive abilities are absolute brain size or neocortex ratio, rather than relative brain size [42,59,60,63,66,67,97]. The consistency of this pattern suggests that absolute measures do in fact tell us something meaningful about cognition (or, alternatively, the motivation) to solve problems. This finding is not limited to primates. The forebrain to hindbrain ratio in birds, which is functionally equivalent to the neocortex ratio in primates, strongly predicts innovation rates [98], while other studies suggest that absolute brain size is a better predictor of performance on cognitive tasks than relative brain size [99]. Although birds have small absolute brain sizes, the high density of neurons in their telencephalon has been used to argue that cognitively sophisticated birds such as corvids and parrots have similar cognitive processing powers to primates [100]. Similarly, a very large number of human neuroscience studies report a relationship between absolute brain (or brain region) size and cognitive performance with no suggestion that there is any need to control for body size [101]. More importantly, a dozen neuroimaging studies from both humans [102–106] and primates [107,108] demonstrate that a substantial neural pathway (the default mode/mentalizing networks involving much of the prefrontal lobe, significant parts of the parietal and temporal lobes, and the limbic system, together with the substantial white matter tracts that connect them, representing a major proportion of the non-visual neocortex [109,110]), correlate with the size of the social group experienced by an individual.

Neuroscientists have specifically addressed the question of how absolute brain size relates to cognitive capacity from both a structural and computational perspective [111]. In primates, these arguments are based on well-established laws of how neuron density scales with brain size [112,113]. Although the hindbrain follows an allometric scaling rule, such that larger bodies require more investment in the physiological and motor control centres, the cortex does not scale linearly with body size [114]—a point originally made, in fact, by Jerison [115]. Moreover, larger brains are structured differently from smaller brains, with greater differentiation [116,117], and a higher density of glial cells that promote transmission efficiency and metabolic efficiency [118]. A complementary argument has been made in evolutionary anthropology: the selection on hominin brain size acted on the cognitive processing power of total brain size, with body size being a linked trait rather than a driver [119].

This bears on the longstanding debate as to whether it is necessary to control for body size in comparative studies of brain evolution. Although many studies automatically control for body size, the rationale for doing so is rarely considered. If it is defended at all, it is usually on the grounds that Jerison [115] did so in his original analyses. However, Jerison's reason for doing so was that larger bodies require more neural architecture devoted to sensory processing, physical coordination, and basic physiological maintenance (e.g. metabolism, thermoregulation, cardiac and respiratory function) and he wanted to remove this so as to focus on the amount of brain matter available for ‘smart’ cognition (in essence, the neocortex). However, including body size in a regression analysis changes the question we ask from one about the correlates of the brain's information processing capacity (a functional, or selection, hypothesis) to one about the correlates of whether a species has a brain that is smaller or larger than would be expected for the average species of a given body size (a developmental question reflecting the costs of growing a large brain) [120]. Perhaps more importantly, it leaves us unable to say whether any relationship is owing to a change in brain size or a change in body size and may say little or nothing about a species’ cognitive potential [121,122]. In fact, it has become increasingly clear in recent years that including body size as a covariate in comparative analyses can result in unpredictable consequences precisely because a radically different question is being asked [123,124]. The lesson is that a great deal more care needs to be taken in formulating hypotheses than is often exercised.

## 5. Conclusion

Much of the debate about primate brain evolution focuses on whether social relationships or ecology best explain patterns of brain size evolution. An important caveat is that the ecological and behavioural traits we typically focus on in these analyses are not ‘evolvable’ (i.e. directly heritable) traits. For example, group size, which is often used as a proxy for sociability, is the outcome of a set of behavioural, cognitive and physiological competencies (such as sociability, tolerance and aggression, and the many neurobiological mechanisms that make these possible) that are embedded in the neurobiology of the brain. Living in a large group involves much more than just managing relationships: large groups are associated with higher competition (which itself leads to larger home ranges and longer day ranges), more complex coordination issues and the potential for cooperation, as well as a more information-rich environment promoting social learning. In effect, group size is simply a proxy for this constellation of demands. That there are no strong evolutionary constraints on group size itself is supported by evidence that group size has a low phylogenetic signal [125], suggesting that it is labile and responds to shifting ecological challenges. Similarly, neither diet nor home range size is the primary unit of selection; they are the outcome of metabolic, cognitive and locomotory adaptations. Thus, we need a better understanding of these mechanistic traits if we are to understand how ecology and physiology impact on brain evolution and, through this, on behavioural complexity.

Finally, the disproportionate evolution in primate brains has occurred in the neocortex and specifically the frontal cortex, a region most closely associated with emotional regulation, social skills and executive function [61]. This in itself suggests that primate brain size is not the result of evolution for a specific task, but rather a generalized response to a range of cognitive challenges. Taking a more systems-based perspective helps to explain why so many different variables correlate with brain size, and makes it possible to place these relationships into a single framework that allows us to see how they articulate with each other. It may also, we suggest, allow us to see relationships that simpler analyses fail to notice—for example, the fact that diet (or foraging skills) exists in an endogenous rachet with brain size, whereas group size (or at least the behaviours that this depends on) is part of a linear causal cascade.

## Data Availability

The data are provided in the electronic supplementary material [126].
